# An Update of Epidemiological Trends in Enzootic Bovine Leukosis in Italy and an Analysis of Risk Factors Associated with Infection Persistence

**DOI:** 10.3390/pathogens14111088

**Published:** 2025-10-24

**Authors:** Cecilia Righi, Carmen Iscaro, Stefano Petrini, Eleonora Scoccia, Silvia Pirani, Alessandro Fiorucci, Roberto Lomolino, Francesco Feliziani

**Affiliations:** 1 National Reference Center for the Study of Retroviruses Related to Infectious Diseases in Ruminants (CEREL), Istituto Zooprofilattico Sperimentale dell’Umbria e delle Marche “Togo Rosati” (IZSUM), Via G. Salvemini, 1, 06126 Perugia, Italy; c.iscaro@izsum.it (C.I.); s.petrini@izsum.it (S.P.); s.pirani@izsum.it (S.P.); f.feliziani@izsum.it (F.F.); 2 Istituto Zooprofilattico Sperimentale dell’Umbria e delle Marche “Togo Rosati”, Via G. Salvemini, 1, 06126 Perugia, Italy; e.scoccia@izsum.it (E.S.); a.fiorucci@izsum.it (A.F.); 3 Ministero della Salute, Viale Giorgio Ribotta, 5, 00144 Rome, Italy; r.lomolino@sanita.it

**Keywords:** BLV, clusters, EBL, epidemiology, eradication, risk factors

## Abstract

In 2017, the Commission Implementing Decision (European Union [EU]) 2017/1910 officially declared Italy to be enzootic bovine leukosis (EBL)-free. The Commission Delegated Regulation (EU) 2020/689 laid criteria to maintain an official disease-free status. While some infection clusters persist in restricted areas, specific measures are being implemented to eliminate pockets of viral persistence. This updated analysis of current data, concerning epidemiological trends of EBL in Italy from January 2022 to December 2024, aimed to analyze the status of infection clusters in terms of risk factors associated with bovine leukemia virus (BLV) seropositivity to evaluate the effectiveness of the eradication measures. Our findings highlighted an improvement in EBL eradication; however, the Latium region lags behind in terms of disease eradication while the situation in Apulia is being resolved. Campania, which has implemented restrictive and consistent health measures, has the lowest prevalence and incidence rates compared with previous years. Identifying and assessing risk factors that favor EBL persistence in infection clusters is essential as is implementing specific measures to eliminate such clusters, thereby enabling disease eradication and the adoption of targeted prevention strategies.

## 1. Introduction

Bovine leukemia virus (BLV) belongs to the *Retroviridae* family, *Orthoretrovirinae* subfamily, and Deltaretrovirus genus. This etiological agent is responsible for a contagious and chronic infection known as enzootic bovine leukosis (EBL) [1].

The BLV be vertically or more usually horizontally transmitted, through direct and indirect exposure of susceptible animals to infected lymphocytes from blood, saliva, nasal secretions, exudates, milk, colostrum and semen [2]. Uncontrolled contact between herds facilitates both direct and indirect BLV transmission through animal movements, communal grazing, and fence line contact. This continuous external infection pressure undermines herd-level measures such as test-and-cull or segregation, making the absence of inter-herd biosecurity one of the main obstacles to effective EBL control and eradication.

Based on these assessments, general preventative and post-infection measures could be: improvement of the level of biosecurity of farms, to avoid communal grazing, adaptation of electronic identification tools (especially in populations reared in the wild), removal of all infection reservoirs, such as stray cattle, that cannot be traced back to any owner, correctly supply the information systems and plan the necessary control actions in a targeted manner and implement training and awareness programs for farmers.

While lethality rates in infected farms are not particularly high (2–5%), EBL infection poses a significant threat to the livestock cattle industry. Economic losses include decreased animal productivity, increased susceptibility to infectious diseases (such as mastitis and skin infections), animal depreciation, costs incurred for eradication or surveillance plans, and international trade restrictions on the import of cattle and animal products from infected areas [3].

EBL infections are distributed worldwide, mainly because of the cattle trade and related products. Moreover, no vaccines against EBL are commercially available.

The first case of EBL was reported in Germany in 1874 and, to date, infections have continued to rise in Argentina, Brazil, Canada, Japan, and the United States [4,5,6,7].

Worldwide, EBL-free countries are located in Oceania (Australia and New Zealand), North Africa (Tunisia), and Asia (Kyrgyzstan and Kazakhstan) [8].

European countries with an EBL-free status include Austria, Belgium, Cyprus, the Czech Republic, Denmark, Estonia, Finland, France, Germany, Ireland, Italy, Latvia, Lithuania, Luxembourg, the Netherlands, Poland, Portugal, Romania, Slovakia, Slovenia, Spain, Sweden, and the United Kingdom (Northern Ireland) [9]. However, outbreaks persist in parts of Eastern Europe, with cases still being reported in Belarus, Bulgaria, Croatia, Greece, Russia, and Ukraine [8,10,11].

Over the last few decades, strict management procedures and new European Union (EU)-level regulations have been implemented to enforce each EU country’s EBL control program and to reduce the prevalence of BLV-infected animals within herds. EU Animal Health legislation was recently updated with the adoption of Commission Regulation (EU) 2016/429 [12], known as the Animal Health Law, which took effect in April 2021, along with the adoption of numerous Commission-delegated and implementation regulations [1]. Italy was officially declared EBL-free in 2017 [13]; however, Italy still has infection-restricted areas subject to specific measures based on the respective 3-year regional plans. These areas are located in the regions of Apulia, Campania, and Latium.

This study aimed to provide an updated analysis of current data in Italy from January 2022 to December 2024 regarding the epidemiological trends of EBL. In particular, we analyzed the status of infection clusters in relation to risk factors associated with BLV seropositivity to evaluate the effectiveness of eradication measures.

## 2. Materials and Methods

### 2.1. Guidelines on Surveillance Measures in the National Territory of Italy

The Italian Ministry of Health, with the support of the National Reference Center for the Study of Retroviruses Related to Infectious Diseases in Ruminants (CEREL), using the Guidelines on Surveillance Measures in the National Territory for the period 2018–2023, has attempted to guarantee the health of national cattle, including buffalo herds. Five years after the EBL-free status was granted, surveillance measures were updated in 2024 in considerably more detail to demonstrate the absence of infection (Guidelines on Surveillance Measures in the National Territory for the Periods 2024–2030), with consideration to the systems of production and to the risk factors identified, as per Regulation EU 2020/689 [14], Annex IV, Part III, Chapter 2, Section 2.

The updated guidelines provided the following details:

1. Implementation of risk-based surveillance activities across the national territory to maintain a disease-free status at community level. This status can be maintained if, in the national territories, at least 99.8% of cattle farms are disease-free, and all cattle aged >24 months are tested for surveillance activities in slaughterhouses. Moreover, all cattle aged >24 months on farms identified as at risk are tested using serological analysis.
2. Implementation of adequate management of BLV infection clusters for disease eradication purposes through epidemiological surveys and a specific 3-year regional plan.
  An infection cluster of EBL is defined as a geographically well-delimited area within the regional territory, in which one or more of the following conditions are present: (i) the prevalence of BLV outbreak is above the threshold of 0.2% (of controllable herds), according to EU regulatory criteria; (ii) epidemiological investigations conducted in BLV outbreak demonstrate the persistence of infection for at least five years; (iii) epidemiological correlations exist among different farms, highlighting the presence of factors that may favor the persistence and circulation of the virus.
  The main regional plan measures for inclusion are: (i) 100% implementation of controls on the affected population, (ii) increasing the frequency of serological tests and lowering the age of the controllable population, (iii) separating infected animals, and (iv) controlling within-cluster movements and stamping out seropositive animals in the last 5 years.
3. Execution of appropriate diagnostic tests for implementing surveillance programs and eradication plans for infection clusters. The official diagnostic tests, listed in Annex III, Section 3, of Delegated Regulation (EU) 2020/689 [14], are serological tests. The ELISA (serum or milk) test is the primary tool for BLV screening programs and follow-up studies, as it offers a high degree of sensitivity and reliability, besides allowing the use of serum pools, while the AGID (serum samples) test guarantees a high degree of specificity, in accordance with the World Organization for Animal Health [15]. In particular, in three Italian infection clusters, official laboratories perform the ELISA test by serum matrix, and in the case of positive results, CEREL also carries out the AGID test. Direct diagnostic tests (polymerase chain reaction [PCR]/real-time PCR assay, and histological examination) from the CEREL are used in addition to serological tests, mainly for testing seropositive animals slaughtered following EBL confirmation.
4. Implementation of disease control measures in establishments in cases of suspected/confirmed BLV.
  In establishments with suspected EBL cases: (i) notify the National Veterinary Information System on Animal Diseases (SIMAN); (ii) conduct epidemiological, clinical, and laboratory surveys; (iii) identify animals with suspected EBL and register them in the National Database (BDN); (iii) apply movement blocks; and (iv) separate animals with suspected EBL.
  In establishments with cases of confirmed BLV: (i) withdrawal of the disease-free status, with overall assessment derived from epidemiological and laboratory survey findings and identification of the animals not registered within 2 days of confirmation; (ii) separation and stamping out of infected animals within 15 days of confirmation; (iii) undertaken periodic checks every 60 days on cattle aged >12 months until at least two consecutive negative tests are recorded, the first after the first 4 months following the stamping out of infected animals and the second 4 months after the first test; (iv) apply movement blocks, except for immediate slaughter; (v) clean and disinfect the infected farms; and (vi) perform official diagnostic tests in farms that have had direct contact with infected animals
5. Compliance with the required information flows regarding the programming and outcomes of the VETINFO platform of the National Platform of Veterinary Information systems [1].

### 2.2. Italian Epidemiological Data Source

For this study, data extracted from VETINFO [16] regarding EBL eradication and surveillance plans, recorded between January 2022 and December 2024, were collected and analyzed to verify the prevalence and incidence trend of EBL infection in Italy. Data were organized into tables, graphs, and maps to simplify and clarify the visualization of EBL outbreaks. An outbreak is defined as the occurrence of one or more epidemiologically related cases of BLV infection within a farm, confirmed through laboratory testing.

### 2.3. Epidemiological Indicators Considered for the Analysis

To evaluate the effectiveness and efficiency of the implemented measures and to analyze infection persistence within clusters, the following information was assessed:

- Number of EBL outbreaks reported in Italy;
- Number of active EBL outbreaks (infection is still present within the affected farm) or eradicated EBL outbreaks (absence of new seropositive animals) in the Italian clusters;
- Prevalence (%) of notified EBL outbreaks on controlled farms (monitored Italian farm);
- Prevalence (%) of seropositive animals on infected farms;
- Annual trends of outbreak prevalence (%) and incidence (%) in the Italian clusters
- Comparison of controlled and controllable farms (planned farms) in affected regions;
- Comparison of controlled and controllable animals (planned animals) in the affected regions and EBL infection clusters;
- Surveillance activities in the establishment of assembly operations (EAO) located in Italian clusters.

### 2.4. Risk Factors Analysis in the National Surveillance System

To effectively monitor and plan risk-based surveillance activities across the entire national territory, the following risk factors were considered, as outlined in the current Guidelines on Surveillance Measures in the National Territory for the Periods 2024–2030. These factors are known to contribute to the persistence of EBL within infection clusters.

- Free-or semi-free-range establishments and/or those practicing common grazing;
- Movement between pastures within each infection cluster region;
- Establishments that registered outbreaks in the last 5 years;
- The length of time registered from confirmation to eradication of cluster outbreaks;
- The length of time from eradication to re-emergence of infection on the same farm;
- Establishments with a suspended EBL-free status in the last 12 months;
- Epidemiological correlations with a confirmed disease case in the last 2 years.

## 3. Results

### 3.1. Italian Epidemiological Data Associated with Assessment Risk Indicators

Sporadic positivity cases were detected in the entire national territory between 1 January 2022 and 31 December 2024. However, infection clusters comprising groups of cases likely linked through direct infection or short transmission chains [17] were observed in three regions, namely, Apulia, Latium, and Campania. Specifically, from 2022 to 2024, in EBL-free regions of the national territory, the EBL prevalence ranged from 0.001% to 0.003%, with the percentage of controlled farms ranging from 90.70% to 92.92% [16]. Conversely, in the clusters, the prevalence was between 0.23% and 0.26%, with the percentage of controlled farms ranging from 87.60% to 97.06%.

**Figure 1:**
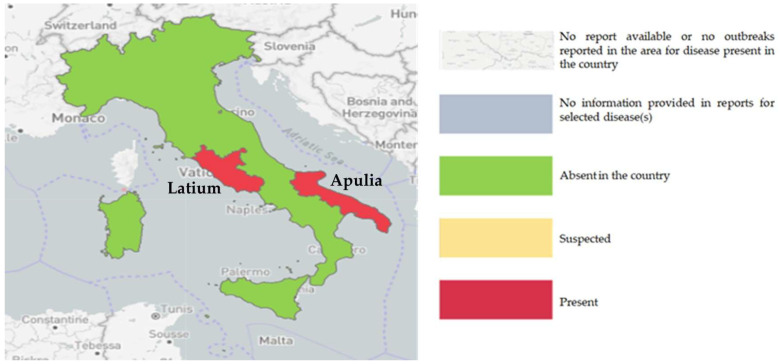
Notified EBL infection areas in Italy in 2024 (SIMAN GIS, updated to 31 December 2024) [10].

Figure 1 shows that, in 2024, EBL infection was located in the central and southern regions, such as Latium and Apulia respectively (clusters). In the Campania region, the cluster remained officially in force, despite the most recent positive case having occurred in November 2021. As no notified outbreak or prevalence/incidence data could be evaluated for the reference period (1 January 2022–31 December 2024), Campania was excluded from the first part of the analysis.

**Figure 2:**
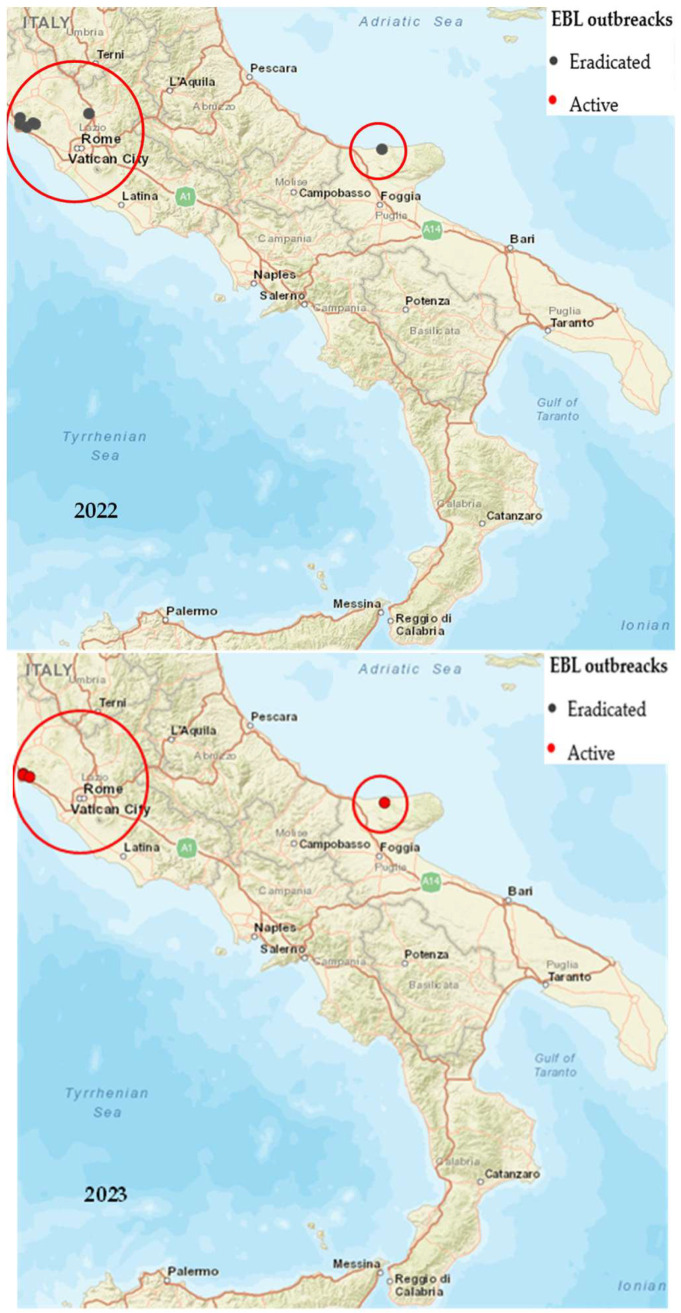

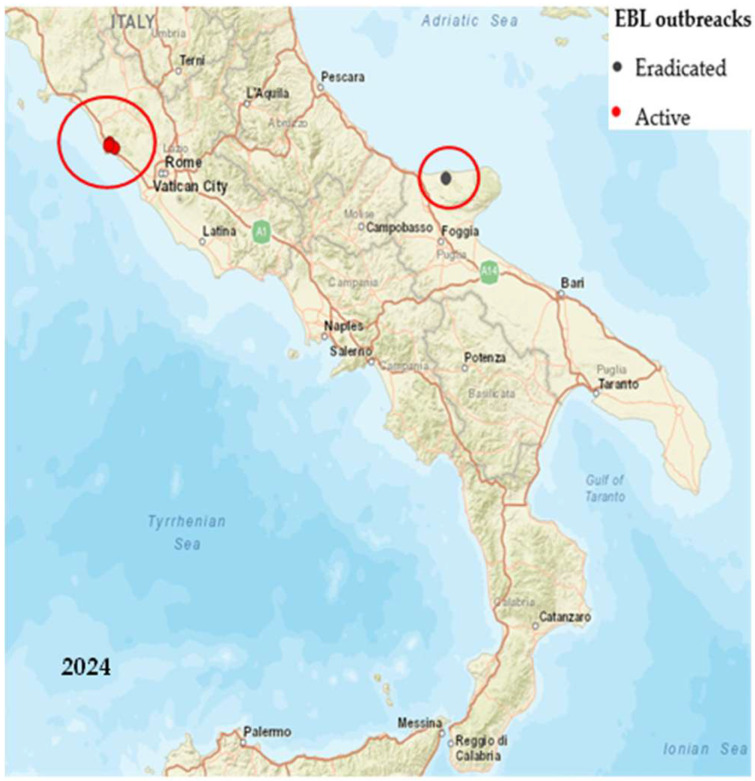
EBL outbreaks notified in Italy for 2022–2024 (SIMAN GIS, updated to 31 December 2024) [10].

In Table 1 and Figure 2, an update on the Italian epidemiological situation is reported based on the number and distribution of EBL outbreaks in Italy recorded from 1 January 2022 to 31 December 2024 [16] (data extraction: 31 December 2024).

Concerning EBL outbreaks examined for bovine species only, it was possible to trace the place and date of suspicion, confirmation, closure, and relapse of the outbreak for each farm. Figure 2 shows infection clusters concentrated in specific restricted areas of the Latium and Apulia regions from 2022 to 2024. Each black point indicates an eradicated outbreak in Italy, recorded from 1 January 2022 to 31 December 2024, while each red point indicates a still active outbreak.

In the Latium region, EBL outbreaks persisted in the Tofa, Santa Marinella, and Allumiere municipalities of the Roma province, and six outbreaks remained active. In the Apulia region, EBL outbreaks had persisted in the Foggia province (specifically in the Lesina and San Nicandro Garganico municipalities) but had been extinguished as at 31 December 2024.

Table 2 shows the annual fluctuations in disease prevalence among notified outbreaks in monitored Italian farms; from 2022 to 2023, the prevalence increased from 0.03% to 0.04%, then decreased again to 0.03% by December 2024.

Table 3 shows similar results concerning the prevalence of EBL seropositive animals in farms, with an increased prevalence from 2022 to 2024.

**Figure 3:**
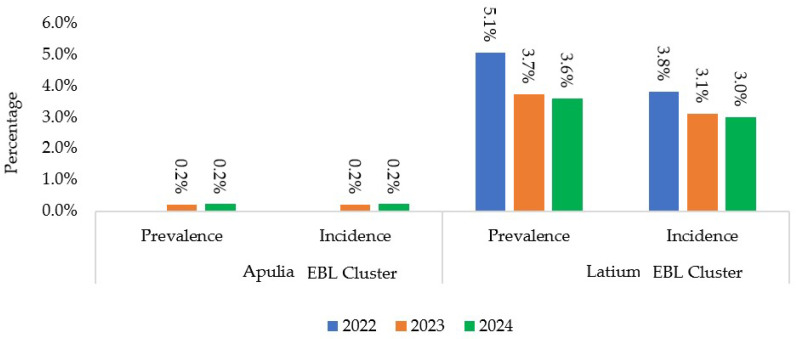
The prevalence and incidence of EBL outbreaks in Apulia and Latium clusters from 2022 to 2024 [16] (updated to 31 December 2024).

The prevalence and incidence of outbreaks in the Apulia and Latium clusters from 2022 to 2024 are shown in Figure 3. Apulia had low prevalence and incidence values (0.2%) and EBL was undetectable in 2022. Despite a decreasing trend, Latium still showed high infection prevalence (proportion of infected animals within a defined population at a given point in time) and incidence (number of new infection cases occurring within a defined population over a specific period of time) rates.

**Figure 4:**
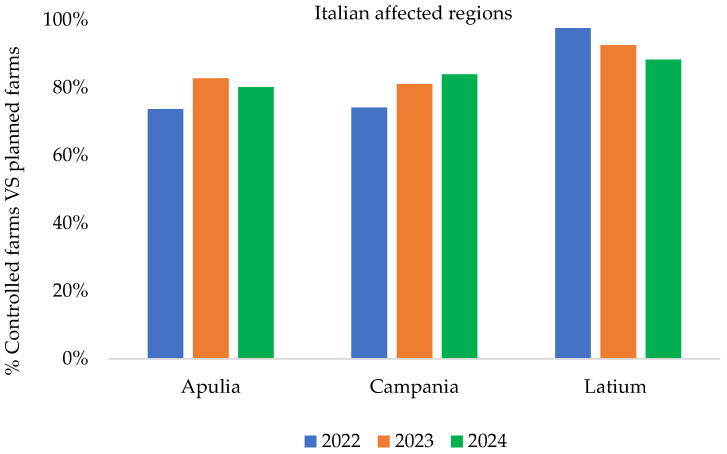
Percentage of controlled vs. planned to be controlled farms in affected regions from 2022 to 2024 [16] (updated to 31 December 2024).

**Figure 5:**
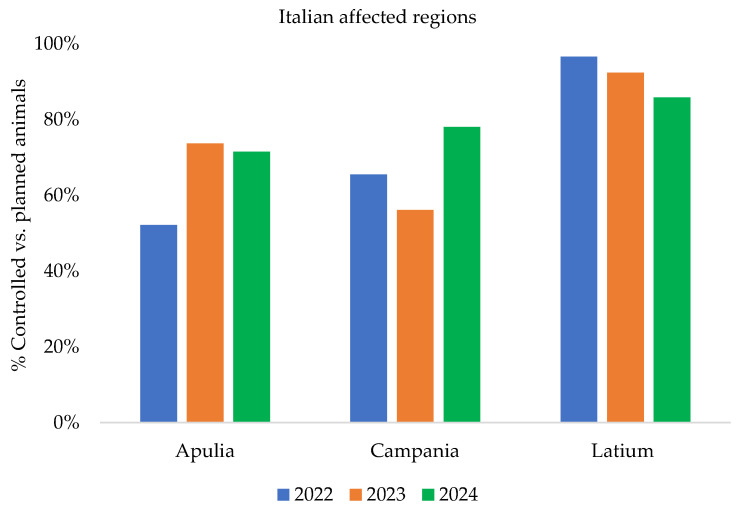
Percentage of controlled vs. planned to be controlled animals in affected regions from 2022 to 2024 [16] (updated to 31 December 2024).

**Figure 6:**
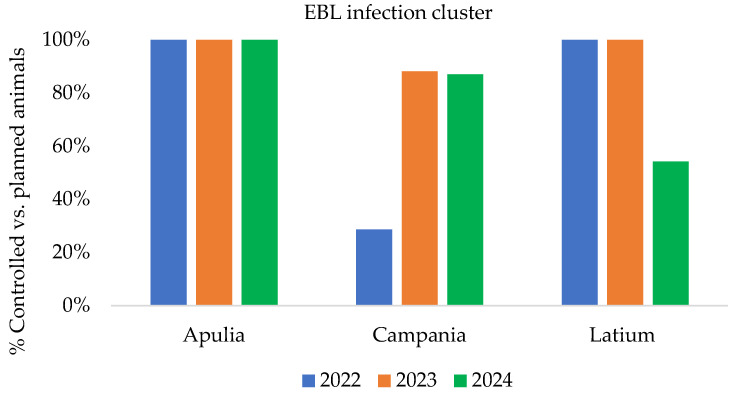
Percentage of controlled vs. planned to be controlled animals in EBL infection clusters from 2022 to 2024 [16] (updated to 31 December 2024).

Figure 4, Figure 5 and Figure 6 show the epidemiological indicators to evaluate the effectiveness and efficiency of the implemented measures and to analyze infection persistence within clusters and entire territory of the affected regions over the years (from 2022 to 2024).

In Figure 4 and Figure 5, data related to the percentage of controlled vs. planned to be controlled farms/animals (ratio between the number of farms or/animals actually controlled and the number of planned farms) were derived from the entire territory of the Apulia, Campania, and Latium regions.

In Figure 6, the percentage of controlled vs. planned to be controlled animal data (ratio between the number of animals actually controlled and the number of planned animals) is derived from EBL clusters in the Apulia, Campania, and Latium regions.

Table 4 shows the EAOs. The EAO are facilities where animals originating from different holdings are gathered for the purpose of trade, transit, or further distribution, before being moved to their final destination. In this way, cattle come into direct contact with animals from other herds, exposing yourself to possible EBL infection and they require serological investigations [16]. Between 2022 and 2024, 55.5–100% of the control activities in the EAOs were performed in the Campania cluster. Conversely, no EAO checks were undertaken for the Apulia and Latium clusters.

### 3.2. Italian Epidemiological Data Associated with Assessment Risk Factors Analysis

**Figure 7:**
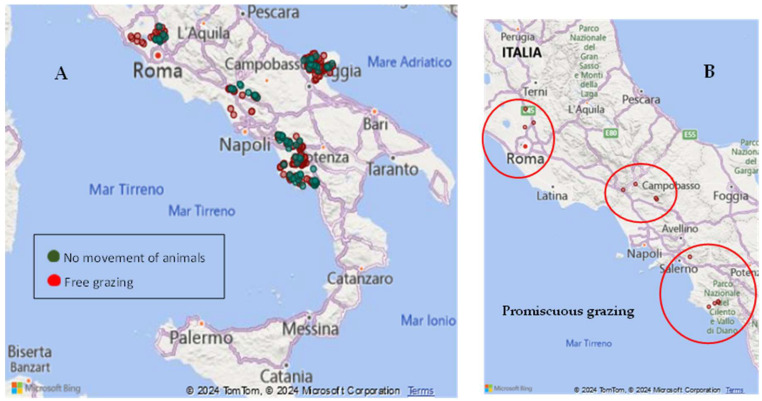
(**A**) Map of the pastures in EBL infection cluster regions; (**B**) Map of the promiscuous grazing in EBL infection clusters [16] (updated to 31 December 2024).

In this section, the potential risk factors for BLV transmission within the Italian cluster regions are examined. Currently, in these clusters, cattle are kept in common grazing areas (i.e., at Tolfa and Allumiere Agrarian Universities), where they can come into contact with feral animals. The maps in Figure 7 A, B show the presence of pastures involving free-range or semi-free-range establishments in terms of EBL infection clusters, and the location of promiscuous grazing in which animals move from different farms and are found together in the same pasture. The data extracted from VETINFO [16] only concerned georeferenced pastures.

Table 5 shows the percentage of pastures with herds moving to other pastures within own EBL cluster region between January 2022 and December 2024. Campania had the highest percentage of pastures with herds that moved within own cluster region.

**Figure 8:**
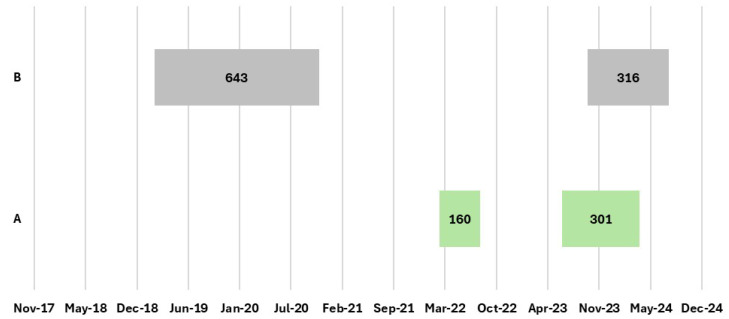
EBL outbreak farms in the Apulia region (A–B), with recurrence in the previous 5 years, reported during the 2022–2024 period. For each outbreak within a farm, the number of days from the confirmation date to the eradication date was recorded [16] (updated to 31 December 2024).

**Figure 9:**
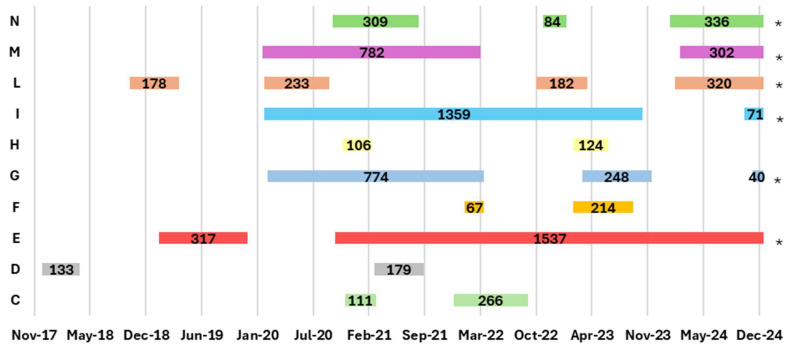
EBL outbreak farms in the Latium region (C–N), with recurrence in the previous 5 years, reported during the 2022–2024 period. For each outbreak within a farm, the number of days from the confirmation date to the eradication date was recorded [16] (updated to 31 December 2024). * Outbreaks still ongoing as of 31 December 2024.

In Table 6 and Figure 8 and Figure 9, an analysis of the previous 5 years was conducted for all outbreak farms with recurrences in the EBL infection clusters reported during the 2022–2024 period. Specifically: (i) for outbreak farms in 2022, data from 2017 to 2022 were analyzed; (ii) for outbreak farms in 2023, data from 2018 to 2023 were analyzed; and (iii) for outbreak farms in 2024, data from 2019 to 2024 were analyzed. For each outbreak on a farm, the duration of the outbreak by calculating the number of days between the date when the infection was officially confirmed from veterinary services (confirmation date) and the date when the farm was declared free of the infection (eradication date), was recorded.

During the considered period (2017–2024), two farms in Apulia (A, B) and 10 farms in Latium (C, D, E, F, G, H, I, L, M, and N) were sites of at least one recurrent EBL outbreak. During this period, in the Latium cluster within the same farm, an outbreak recurred at a minimum of two to a maximum of four times. Furthermore, for two outbreak farms (E and I), the period from confirmation to 31 December 2024 was approximately 4 years, with these outbreaks still ongoing at this date.

Table 7 shows, in cluster regions, the number of farms in which EBL infection persisted within the same farm in the last 5 years.

In the last 12 months, the EBL-free status was suspended in six farms in the Latium region (E, G, I, L, M, and N). Moreover, in the last two years (2022–2024), in nine of 10 farms in Latium (C, E, F, G, H, I, L, M, and N) that were sites of at least one EBL outbreak, epidemiological correlations with a confirmed disease case were reported (Table 6 and Table 7, Figure 9).

In the Campania cluster, the latest positive case occurred in November 2021. From 2017 to 2021, in that same farm, no outbreak recurred in the last 5 years or the EBL-free status was suspended in the preceding 12 months, consequently resulting in no epidemiological links (Table 6 and Table 7, Figure 9).

In the Apulia cluster, regarding the two farm outbreaks (A, B) in which EBL infection recurred in the last 5 years (2017–2024), the EBL-free status preceding 12 months was suspended, but there was only an epidemiological link within the same farm in the last 2 years (Table 6 and Table 7, Figure 9).

## 4. Discussion

EBL is the most frequent tumor disease in cattle, but it can go unnoticed clinically because a high percentage of BLV-infected animals are asymptomatic [18]. Nevertheless, recent serological investigations have shown an increase in the infection rates in several countries, including Argentina [19], Brazil [20], Canada, Japan, and the United States [8]. Despite being granted an EBL-free status in 2017 [13], Italy’s eradication program has not been fully successful in some regions. Efforts and financial resources have been directed into these regions where EBL eradication has proved to be more challenging.

Numerous control-specific measures have been undertaken [21] over recent years, following Guidelines on National Territory Surveillance Measures for 2018–2023 updated for 2024–2030, to reduce the effect of EBL on herds and decrease the prevalence of BLV-infected animals within herds. These measures have also included efforts to identify and assess epidemiological indicators as well as risk factors favoring EBL persistence in infection clusters to enable disease control and the adoption of potential prevention strategies.

In Italian affected regions, serodiagnosis is performed to control the spread of the infection and to remove infected animals. The ELISA test serves as the primary tool for BLV screening programs and follow-up studies due to its high sensitivity, while the AGID test provides a high degree of specificity, in accordance with the recommendations of the World Organisation for Animal Health [15].

However, the PCR assay from CEREL is used in addition to serological tests, mainly to test seropositive animals slaughtered following EBL confirmation.

This study provides a general overview of the epidemiological trend of EBL in Italy over the 3-year period from 1 January 2022 through 31 December 2024, focusing on the analysis and assessment of risk indicators and factors favoring EBL persistence, which are likely to be useful for enhancing EBL control and mitigating possible risks.

Our results showed a steady trend in the prevalence rate of BLV in Italy during the period from 1 January 2022 to 31 December 2024 compared with the previous 3 years (2018–2021) [1], with a mean herd EBL prevalence rate of 0.03% and a reduction in the total number of EBL outbreaks reported in Italy (from 34 to 23).

As expected, in a nationwide analysis of data concerning EBL-free territories from 2022 to 2024, seropositivity rates were lower (0.001–0.003%) than the EBL prevalence in the cluster regions (0.23–0.26%), demonstrating that EBL prevalence was concentrated in specific areas (Apulia, Campania, and Latium), with persistence in some factors responsible for delaying the eradication process.

However, the Campania region, initially included as a cluster region [1], subsequently adopted stringent measures and had its last outbreak of infection in November 2021, and currently has the appropriate conditions to request and obtain an EBL-free status. In contrast, the Apulia and Latium regions lag behind in terms of being able to obtain an EBL-free status.

In the Apulia region, constant prevalence and incidence rates of infection (0.02%) were registered between 2022 and 2024, reflecting the trend observed during the 2018–2021 period [1]. Clusters persisted in the municipalities of Lesina and San Nicandro Garganico, where infection had also re-emerged after long periods (2 years) of apparent absence.

In the Latium region, the eradication program has been unsuccessful in eradicating infections in some municipalities, with infections recurring several times over the years. In the cluster areas, high infection prevalence (3.6–5.1%) and incidence (3–3.8%) rates were registered between 2022 and 2024. However, an overall decrease in annual prevalence and incidence rates was noted from 2022 to 2024, indicating continuous, albeit slow, improvements.

We investigated factors that influence the persistence and duration of outbreaks, and highlight strategies that can be employed to mitigate outbreak durations. The duration of an outbreak varies depending on factors such as the type of disease, environmental conditions, and effectiveness of public health interventions. Challenges in managing EBL stem from complex aspects such as the long incubation period from infection to onset and its slow spread, which makes it difficult to accurately understand disease progression [8]. Moreover, in local geographical situations, such as the Italian clusters, some outbreaks persist as a result of various well-known territory-related challenges. For example, the presence of indocile or feral cattle makes it difficult to perform constant and complete checks in terms of health parameters, and the presence of unidentified animals not referable to any owner under conditions of promiscuity with reared cattle representing the most important reservoir of BLV [1].

In this study, with reference to the Guidelines on National Territory Surveillance Measures for 2018–2023 (updated for 2024–2030) and relevant indicators identified in the literature [22], we analyzed certain risk factors potentially associated with seropositivity in the infected farms to clarify their role in the presence of BLV infection.

Data for each cluster were collected in relation to farm and animal monitoring, timing of the outbreak, transhumance and grazing management, and EAO checks. A potential risk factor for the introduction of the BLV into herds is the movement of infected animals into free-grazing areas [22], which is often associated with promiscuity. Stocks grazing away from the main farm area can be presumed to be more at risk of being exposed to BLV because of their physical contact with animals from other herds. Where animals could not be moved from areas of infection clusters, a measure was implemented requiring notification in situations where free grazing cattle were being kept in university or agricultural community pastures (i.e., the Tolfa and Allumiere Agrarian Universities) within the same territories to prevent all unauthorized and illegal movements; however, this still allowed for promiscuity.

In the persistent clusters, we observed a low percentage of pastures in which herds were moved to other pastures within their EBL cluster regions. The Campania cluster, which was EBL-free, had the highest percentage in this regard. Thus, this condition did not appear to be associated with BLV circulation in our study.

As expected, the movement of infected animals into free grazing areas represents a potential risk for BLV infection; a plausible explanation for this finding may be the lack of data registration on the VETINFO platform. Occasionally, veterinary information systems [16] are not fully completed or updated regularly and, in some cases, data are unavailable or incomplete.

Another potential risk for BLV transmission is the lack of EAO checks [1]. The enhanced EAO control activities in the Campania region compared with those in Latium and Apulia regions likely contributed to achieving the appropriate conditions necessary to request and obtain an EBL-free status for Campania, emphasizing the importance of surveillance measures.

We provided findings concerning the persistence of recurring outbreaks, the timing of the outbreaks, and epidemiological correlations between outbreaks within the same farm. These findings strongly highlight the need to eradicate outbreaks as quickly as possible to reduce the risk of further transmission to surrounding areas.

In Apulia, the duration of an outbreak varied from 160 days to >21 months, whereas in Latium, outbreaks lasted from 40 days to >2 years, exceeding the expected maximum period allowed to reacquire an EBL-free status (Figure 8 and Figure 9).

Moreover, multiple recurring outbreaks were observed in both regions on the same farm, often with epidemiological links. Between 2022 and 2024, when analyzing outbreaks on a farm-by-farm basis over the previous 5 years (2017–2024), there was a 30% recurrence rate (10 of 34 farms) in Latium and 11% (2 of 18 farms) in Apulia. Most of these recurrent outbreaks showed epidemiological correlations within the same farm in the past 2 years (9 of 10 in Latium, 1 of 2 in Apulia), highlighting the continued challenges in eradicating BLV from these areas.

Stricter control measures have been implemented in the identified territorial clusters; however, their application remains difficult, often because of political, cultural, and socioeconomic constraints, and a lack of sufficient resources. Moreover, in some cases, farmers often struggle to understand the epidemiological complexity of a chronic disease that is mostly asymptomatic, and which may re-emerge after extended periods of apparent absence within the same farm. Consequently, their collaboration with veterinary services in enforcing control measures is not always active.

These conditions facilitate illegal animal exchange and inconsistent implementation of biosecurity measures, hindering the effective interruption of transmission chains.

In contrast, in other regions, farmers are highly cooperative, as they recognize the value of the required measures aimed at improving the health status of their herds.

This study’s findings indicate that there has been a continued improvement in the Italian epidemiological situation regarding EBL clusters, but enhanced surveillance is necessary in these cluster areas to detect any potential disease recurrence. Currently, the prevalence is moderate, in which case the most effective strategy for controlling EBL involves identifying and eliminating infected animals (‘test and remove’), and identifying risk factors that predispose to infection. Simultaneously, an adequate compensation system would be helpful for farmers who incur disease-related damage or losses. Most European countries have successfully employed this method to control EBL despite it being expensive [8].

The “test and remove” strategy is mandated by the national surveillance and eradication plan for EBL and is uniformly applied across the entire Italian territory whenever a positive animal is detected on a farm which must be removed. In addition, in Italy, a compensation scheme is in place to cover both direct and indirect losses arising from the occurrence of epidemic diseases in livestock holdings, including EBL.

This study has some limitations. As with most observational studies, our approach provides a simple and practical yet basic framework for estimating potential risk indicators for BLV introduction. More refined multivariate analyses are necessary to further clarify the associations between these risk factors and EBL persistence, including the exploration of additional variables (e.g., rearing method, and herd size, age, and range).

The sampling system used in this study reflected the cattle population collected in VETINFO [16] with acceptable accuracy but it is subject to some level of uncertainty owing to a lack of systematic documentation, such as cattle registration, promiscuous breeding of herds in some municipalities, and unrecorded animal movements.

Future efforts should include the continued application of the ‘test and remove’ strategy to eliminate infected animals and the systematic monitoring of infection prevalence over time. In parallel, further research is needed to achieve a comprehensive understanding of the interaction between BLV and associated risk factors, in order to implement targeted control measures to address the underlying causes of EBL persistence and, crucially, to promote a significant shift in farmers’ mindsets toward disease awareness, biosecurity, and active participation in eradication programs in specific areas.

## Figures and Tables

**Table 1:** Number of EBL-notified outbreaks in Italy (in EBL-free territories and clusters) for 2022–2024 [16] (updated to 31 December 2024).

Years	Number of EBL Outbreaks			
	North Italy	Central Italy	South Italy	Total
2022	0	6	1	7
2023	0	6	3	9
2024	0	6	1	7
**Total**	**0**	**18**	**5**	**23**

**Table 2:** Prevalence of notified EBL outbreaks in controlled cattle and water buffalo farms per year (in EBL-free territories and clusters) between January 2022 and December 2024 [16] (updated to 31 December 2024).

Years	No. of Controlled Farms	Total No. of EBL Outbreaks		Prevalence of EBL Outbreaks		Total Prevalence of EBL Outbreaks
		Apulia	Latium	Apulia	Latium	
2022	21.095	1	6	0.004%	0.02%	0.03%
2023	19.770	3	6	0.01%	0.03%	0.04%
2024	18.277	1	6	0.005%	0.03%	0.03%

**Table 3:** Prevalence of EBL seropositive animals (cattle and water buffalo) in infected farms (in EBL-free territories and clusters) between 2022 and 2024 [16] (updated to 31 December 2024).

Years	Animals Controlled	Seropositive Animals		Prevalence of EBL-Seropositive Animals		Total Prevalence of EBL- Seropositive Animals
		Apulia	Latium	Apulia	Latium	
2022	509.400	2	8	0.0003%	0.001%	0.001%
2023	569.300	6	7	0.001%	0.001%	0.002%
2024	550.753	4	8	0.0007%	0.001%	0.002%

**Table 4:** EBL eradication plan surveillance activities in the EAOs in persistent clusters [16] (updated to 31 December 2024).

Years	Region	No. of EAOs Under the Eradication Plan	No. of Checked EAOs	% EAO Coverage
2022	Apulia	0	0	0%
	Campania	10	9	90%
	Latium	1	0	0%
2023	Apulia	0	0	0%
	Campania	9	5	55.5%
	Latium	1	0	0%
2024	Apulia	0	0	0%
	Campania	6	6	100%
	Latium	0	0	0%

**Table 5:** Percentage of pastures with movements of herds to other pastures within the own EBL cluster region between January 2022 and December 2024 [16] (updated to 31 December 2024).

Years	Clusters Regions	Percentage of Pastures with Herds’ Movements Within the Own EBL Cluster Region
2022	Apulia	17.6%
	Campania	23.2%
	Latium	15.7%
2023	Apulia	13.6%
	Campania	21.5%
	Latium	16.9%
2024	Apulia	12.6%
	Campania	22%
	Latium	18.3%

**Table 6:** Outbreak farms in the EBL cluster regions, with recurrence in the previous 5 years, reported during the 2022–2024 period [16] (updated to 31 December 2024).

Years	EBL Outbreaks Farms in the EBL Cluster Regions with Recurrence in the Previous 5 Years		
	Apulia *	Campania	Latium **
**2017**	-	-	D
**2018**	-	-	D, L
**2019**	B	-	E, L
**2020**	B	-	C, E, G, H, I, L, M, N
**2021**	-	-	C, D, G, H, I, M, N
**2022**	A	-	C, F, G, H, I, L, M, N
**2023**	A, B	-	F, G, H, I, L, N
**2024**	A, B	-	G, I, L, M, N

**Apulia** * (A, B): farms with outbreaks eradicated as of 31 December 2024. **Latium** ** (C, D, F): farms with outbreaks eradicated as of 31 December 2024; (E, G, I, L, M, N): farms with outbreaks still ongoing as of 31 December 2024.

**Table 7:** Persistence of EBL infection in cluster regions within the same farm: number of establishments that registered recurring outbreaks in the last 5 years (2017–2024), number of establishments with suspended EBL-free status in the last 12 months (from 31 December 2023 to 31 December 2024), and the number of epidemiological correlations with a confirmed disease case in the last 2 years [16] (updated to 31 December 2024).

Clusters Regions	No. of Farms with EBL Outbreaks in the Last 5 Years	No. of Farms With EBL-Free Status Suspended in the Last 12 Months	No. of Epidemiological Links with Confirmed EBL Case in the Last 2 Years
Apulia	2	2	1
Campania	0	0	0
Latium	10	6	9

## Data Availability

The original contributions presented in this study are included in the article. Further inquiries can be directed to the corresponding author.

## References

[1] Righi C., Iscaro C., Petrini S., Lomolino R., Feliziani F. (2021). Enzootic bovine leukosis in Italy: Epidemiological issues after free status recognition and measures applied to tackle the last persistent clusters. Pathogens.

[2] Benitez O.J., Roberts J.N., Norby B., Bartlett P.C., Takeshima S.N., Watanuki S., Aida Y., Grooms D.L. (2019). Breeding bulls as a potential source of bovine leukemia virus transmission in beef herds. JAVMA J. Am. Vet. Med. Assoc..

[3] Ott S.L., Johnson R., Wells S.J. (2003). Association between bovine-leukosis virus seroprevalence and herd-level productivity on US dairy farms. Prev. Vet. Med..

[4] Nekouei O., Vanleeuwen J., Sanchez J., Kelton D., Tiwari A., Keefe G. (2015). Herd-level risk factors for infection with bovineleukemia virus in Canadian dairy herds. Prev. Vet. Med..

[5] Murakami K., Kobayashi S., Konishi M., Kameyama K.-I., Tsutsui T. (2013). Nationwide survey of bovine leukemia virus infection among dairy and beef breeding cattle in Japan from 2009–2011. J. Veter. Med. Sci..

[6] Ma B., Gong Q., Sheng C., Liu Y., Ge G., Li D., Diao N., Shi K., Li J., Sun Z. (2021). Prevalence of bovine leukemia in 1983–2019 in China: A systematic review and meta-analysis. Microb. Pathog..

[7] Ladronka R.M., Ainsworth S., Wilkins M.J., Norby B., Byrem T.M., Bartlett P.C. (2018). Prevalence of bovine leukemia virus antibodies in US dairy cattle. Vet. Med. Int..

[8] Lv G., Wang J., Lian S., Wang H., Wu R. (2024). The global epidemiology of bovine leukemia virus: Current trends and future implications. Animals.

[9] Commission Implementing Regulation (EU) 2021/620 of 15 April 2021 Laying Down Rules for the Application of Regulation (EU) 2016/429 of the European Parliament and of the Council as Regards the Approval of the Disease-Free and Non-Vaccination Status of Certain Member States or Zones or Compartments thereof as Regards Certain Listed Diseases and the Approval of Eradication Programmes for Those Listed Diseases. http://data.europa.eu/eli/reg_impl/2021/620/oj.

[10] OIE Platform WAHIS. https://wahis.oie.int/#/dashboards/country-or-disease-dashboard.

[11] EFSA AHAW Panel (European Food Safety Authority Panel on Animal Health and Welfare) (2015). Enzootic bovine leukosis. EFSA J..

[12] Commission Regulation (EU) 2016/429 of the European Parliament and of the Council of 9 March 2016 on Transmissible Animal Diseases and Amending and Repealing Certain Acts in the Area of Animal Health (‘Animal Health Law’). http://data.europa.eu/eli/reg/2016/429/oj.

[13] Commission Implementing Decision (EU) 2017/1910 of 17 October 2017 Amending Decision 93/52/EEC as Regards the Brucellosis (*B. melitensis*)-Free Status of Certain Regions of Spain, Decision 2003/467/EC as Regards the Official Bovine Brucellosis-Free Status of Cyprus and of Certain Regions of Spain, and as Regards the Official Enzootic-Bovine-Leucosis-Free Status of Italy, and Decision 2005/779/EC as Regards the Swine Vesicular Disease-Free Status of the Region of Campania of Italy. http://data.europa.eu/eli/dec_impl/2017/1910/oj.

[14] Commission Delegated Regulation (EU) 2020/689 of 17 December 2019 Supplementing Regulation (EU) 2016/429 of the European Parliament and of the Council as Regards Rules for Surveillance, Eradication Programmes, and Disease-Free Status for Certain Listed and Emerging Diseases. http://data.europa.eu/eli/reg_del/2020/689/oj.

[15] World Organization for Animal Health (2018). Chapter 3.4.9 Enzootic bovine leukosis. Manual of Diagnostic Tests and Vaccines for Terrestrial Animals.

[16] VETINFO. https://www.vetinfo.it/.

[17] Miquel S., Porta and International Epidemiological Association (2008). A Dictionary of Epidemiology.

[18] Lancheros-Buitrago D.J., Bulla-Castañeda D.M., Giraldo-Forero J.C., Pulido-Medellin M.O. (2023). Risk factors associated with enzootic bovine leukosis in Boyacá and Cundinamarca municipalities, Colombia. Open Vet. J..

[19] Panei C.J., Pérez Aguirreburualde M.S., Echeverría M.G., Galosi C.M., Torres A., Silva H.J.E. (2017). Seroprevalencia de infección por el virus de leucosis bovina durante 2015 en rodeos de críade la Zona Deprimida del Río Salado, provincia de Buenos Aires, Argentina. Analecta Vet..

[20] Ramalho G.C., Silva M.L.C.R., Falcão B.M.R., Limeira C.H., Nogueira D.B., Dos Santos A.M., Martins C.M., Alves C.J., Clementino I.J., Santos C.D.S.A. (2021). High herd-level seroprevalence and associated factors for bovine leukemia virus in the semi-arid Paraíba state, Northeast Region of Brazil. Prev. Vet. Med..

[21] Ordinanza Ministeriale 28 Maggio 2015, Misure Straordinarie di Polizia Veterinaria in Materia di Tubercolosi, Brucellosi Bovina e Bufalina, Brucellosi Ovi-Caprina, Leucosi Bovina Enzootica. Gazzetta Ufficiale Serie Generale n.144, 24 June 2015. https://www.gazzettaufficiale.it/eli/id/2015/06/24/15A04879/sg.

[22] Selim A., Manaa E.A., Alanazi A.D., Alyousif M.S. (2021). Seroprevalence, risk factors and molecular identification of bovine leukemia virus in Egyptian cattle. Animals.

